# Drug-Induced Toxic Epidermal Necrolysis: A Case Report

**DOI:** 10.7759/cureus.89368

**Published:** 2025-08-04

**Authors:** Tânia Pereira da Silva, Maria Luis Santos, Ana Patrícia Brito, Magda Fernandes, Jorge Cotter

**Affiliations:** 1 Internal Medicine, Unidade Local de Saúde do Alto Ave, Guimarães, PRT

**Keywords:** dermatological emergency, drug reaction, ibuprofen, nsaids, toxic epidermal necrolysis

## Abstract

Toxic epidermal necrolysis (TEN) is a rare, life-threatening mucocutaneous condition, most commonly triggered by drugs, with particularly high mortality in elderly populations. The medications most frequently associated with TEN include antibiotics such as sulfamethoxazole-trimethoprim (sulfonamides), penicillins, cephalosporins, and quinolones (e.g., ciprofloxacin); anticonvulsants such as phenytoin, carbamazepine, and lamotrigine; nonsteroidal anti-inflammatory drugs (NSAIDs), especially oxicam derivatives (e.g., piroxicam), and, less commonly, ibuprofen; allopurinol; nevirapine; and other agents such as paracetamol, celecoxib, tyrosine kinase inhibitors, and immunobiological therapies like infliximab.

We describe a 71-year-old, functionally dependent male who developed fever and progressive skin detachment after self-medicating with paracetamol and ibuprofen. Examination revealed widespread bullae and skin sloughing involving approximately 70% of his total body surface area (TBSA). Despite supportive measures, the patient rapidly deteriorated and died on the second day of hospitalization. TEN was assumed as the primary cause of death.

The prognosis in TEN is strongly influenced by age, comorbidities, and the extent of skin involvement. Genetic predispositions, such as HLA-B*15:02 *(associated with carbamazepine-induced TEN, particularly in Southeast Asian populations), HLA-A31:01 (carbamazepine hypersensitivity in European, Japanese, and Latin American populations), and HLA-B*58:01 (allopurinol-induced TEN in Asian populations), are important risk factors that guide genetic screening recommendations before prescribing certain high-risk drugs.

Supportive care remains the mainstay of treatment for TEN, focusing on meticulous wound care, fluid and electrolyte management, infection prevention, and pain control. However, the use of immunomodulatory therapies, such as corticosteroids, cyclosporine, and biologics, remains a subject of ongoing debate, with inconsistent evidence regarding their efficacy.

Timely recognition of TEN and immediate cessation of the offending drug are paramount to improving patient outcomes and reducing mortality.

This case highlights the potential role of ibuprofen, a commonly used over-the-counter NSAID, as a trigger for TEN, particularly in frail elderly individuals. Although ibuprofen is generally considered a low-risk medication, its capacity to induce severe cutaneous adverse reactions should not be underestimated. The prognosis in TEN is heavily influenced by factors such as advanced age, existing comorbidities, and the extent of skin involvement.

Given the high fatality rates associated with TEN, especially in older adults, clinicians should maintain a high index of suspicion in patients presenting with acute mucocutaneous symptoms following recent drug exposure, including over-the-counter medications like ibuprofen, and emphasize the importance of public education on the dangers of unsupervised self-medication.

## Introduction

Toxic epidermal necrolysis (TEN), also known as Lyell’s syndrome, is a severe mucocutaneous hypersensitivity reaction characterized by extensive keratinocyte apoptosis and full-thickness epidermal necrosis, leading to widespread skin detachment. It represents the most severe end of the Stevens-Johnson Syndrome (SJS)/TEN spectrum and is defined by epidermal involvement of more than 30% of the total body surface area (TBSA) [1].

Drugs are the most common triggers of TEN, with anticonvulsants (e.g., carbamazepine, phenytoin), sulfonamide antibiotics, allopurinol, and nonsteroidal anti-inflammatory drugs (NSAIDs), particularly oxicam derivatives, being most frequently implicated. Although traditionally considered a low-risk NSAID, ibuprofen has been increasingly reported as a causative agent, particularly in vulnerable populations such as the elderly [2,3]. Recent case reports have underscored the underestimated role of widely used over-the-counter medications in triggering this devastating condition [4].

Immunologically, TEN is classified as a type IVc delayed hypersensitivity reaction involving cytotoxic T lymphocytes and natural killer (NK) cells. These immune effector cells mediate keratinocyte apoptosis via the release of perforin, granzyme B, tumor necrosis factor-alpha (TNF-α), and granulysin, the latter being a key molecule in widespread epidermal destruction [5,6].

Risk factors for TEN include advanced age, female sex, specific genetic predispositions (e.g., HLA-B15:02, HLA-A31:01, HLA-B*58:01), and underlying chronic conditions such as renal failure or malignancy [7]. Mortality rates exceed 30% in many case series and are particularly high in elderly patients with comorbidities. The SCORTEN (SCORe of Toxic Epidermal Necrosis) index remains a widely accepted prognostic tool [8].

This report describes a fatal case of TEN, presumably induced by ibuprofen, highlighting the need for early recognition, immediate withdrawal of the offending agent, and patient education regarding the risks of unsupervised medication use.

## Case presentation

**Figure 1:**
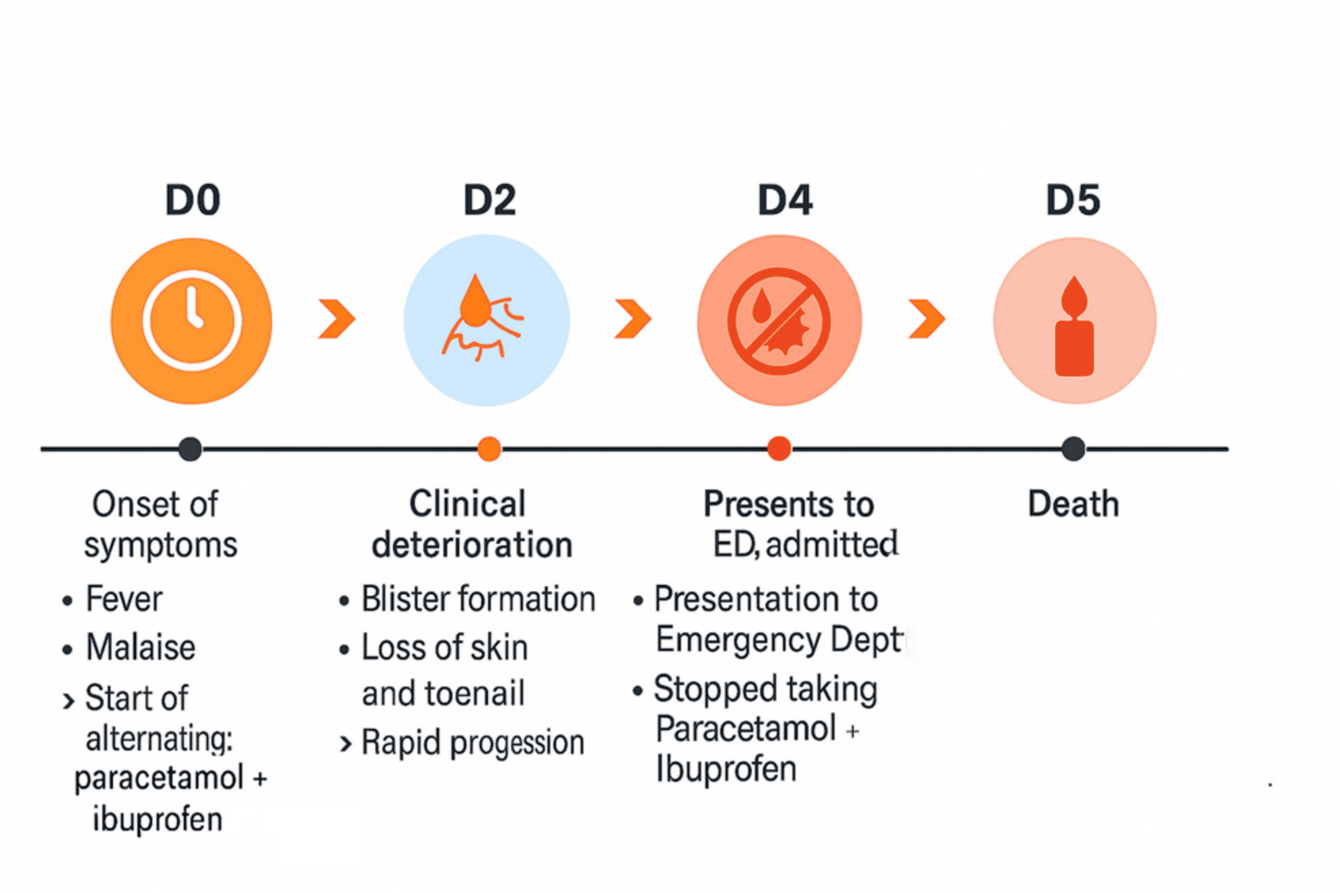
Temporal clinical evolution of the patient’s condition over the course of the illness

A 71-year-old man, functionally dependent, presented to the emergency department with generalized weakness, moaning, and a one-week history of fever. During this period, he self-medicated with alternating doses of paracetamol (1 g) and ibuprofen (400 mg) every eight hours in response to febrile episodes, with a maximum recorded temperature of 39 °C. These medications were taken continuously until hospital admission. The patient remained febrile throughout, with a persistently elevated temperature profile exceeding 38 °C despite antipyretic use. Two days after symptom onset, he developed painful bullous eruptions followed by rapid epidermal detachment involving the limbs, trunk, back, and posterior scrotum, affecting approximately 70% of total body surface area (TBSA). The temporal clinical evolution is outlined in Figure 1.

Physical examination revealed a positive Nikolsky sign. Mucosal surfaces were spared.

**Figure 2:**
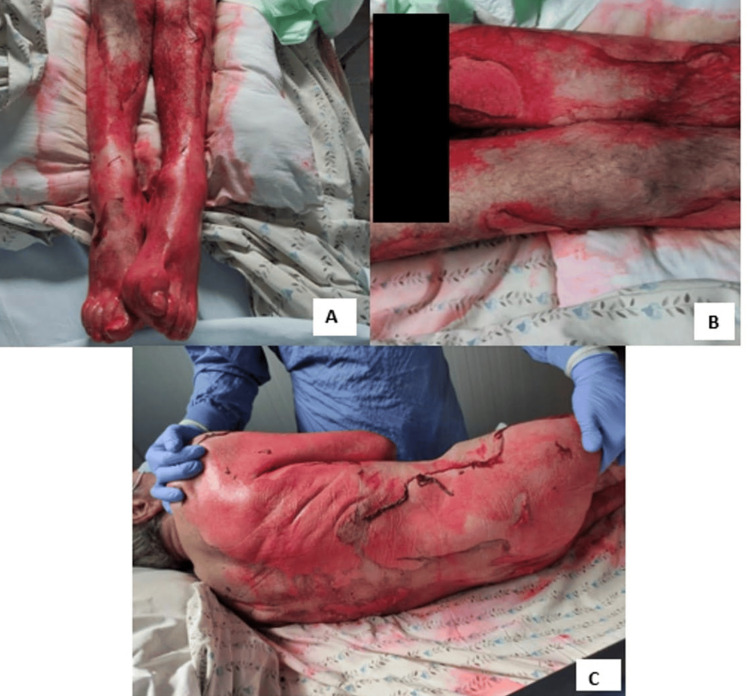
Lyell's syndrome Images A and B show epidermal detachment on the lower limbs, while image C shows detachment on the back, characteristic of toxic epidermal necrolysis.

Given the extent of cutaneous involvement (Figure 2) and the patient's baseline frailty, the burns unit was consulted, and a comfort-focused treatment plan was adopted. Supportive measures included fluid resuscitation, topical antiseptics, and analgesia. Despite these interventions, the patient's condition deteriorated rapidly, and he died on the second day of hospitalization.

## Discussion

TEN is a dermatological emergency frequently triggered by commonly used medications. Although historically considered low risk, NSAIDs such as ibuprofen have been increasingly implicated in recent literature, particularly among elderly individuals and patients with multiple comorbidities [2,3,9].

Prodromal symptoms, including fever, malaise, and sore throat, often precede the cutaneous manifestations. These evolve into painful erythematous macules, bullae, and skin detachment, with a positive Nikolsky sign. Mucosal involvement is observed in more than 90% of cases, but was absent in this patient, a finding not inconsistent with the diagnosis [10].

The association between ibuprofen and TEN, although uncommon, has been confirmed in population-based studies, including the EuroSCAR project, which identified an increased risk, particularly in older adults and females [2]. The typical latency period between drug exposure and symptom onset ranges from 4 to 28 days, consistent with this case [11].

Prognostic evaluation using the SCORTEN index (SCORe of Toxic Epidermal Necrosis) is essential for risk stratification and therapeutic planning. This validated tool estimates mortality based on seven clinical and laboratory parameters at admission: age > 40 years, presence of malignancy, heart rate > 120 bpm, epidermal detachment > 10% of total body surface area, serum urea > 10 mmol/L, serum glucose > 14 mmol/L, and serum bicarbonate < 20 mmol/L. Each variable scores 1 point, and mortality increases progressively with the total score. In this case: age > 40 years, skin detachment > 10%, serum urea > 10 mmol/L (~28 mg/dL), metabolic acidosis (serum bicarbonate < 20 mmol/L), along with leukopenia (3,300 leukocytes) and significant lymphopenia (300 lymphocytes) on admission, multiple unfavorable prognostic markers were present, although SCORTEN was not formally calculated at the time [8].

More recently, the CRISTEN (Clinical Risk Score for TEN) score has been proposed as a complementary tool for risk stratification in TEN [12]. It incorporates criteria similar to SCORTEN, with updated clinical and laboratory indicators, including acute kidney injury. In this case, the CRISTEN score was 3 points, indicating an intermediate-to-high risk of mortality, based on: advanced age (71 years), extensive epidermal detachment (70%), and acute renal failure on admission (creatinine 3 mg/dL).

Supportive care remains the cornerstone of treatment, including fluid replacement, infection prevention, pain management, and nutritional support, ideally delivered in specialized intensive care or burn units [13]. Among immunomodulatory therapies, cyclosporine has demonstrated mortality benefit in recent meta-analyses, with faster re-epithelialization and lesion stabilization [11]. Additionally, recent studies, such as Zhang et al. (2022), have evaluated combination therapy with etanercept and systemic corticosteroids, showing promising efficacy in reducing mortality and improving clinical outcomes, although randomized trials are still needed to confirm this indication [14]. Conversely, the roles of intravenous immunoglobulin (IVIG) and TNF-α antagonists remain controversial, requiring individualized decision-making based on clinical presentation [10].

This case highlights the underestimated potential of ibuprofen to cause TEN, particularly in vulnerable individuals. Community education regarding the risks of over-the-counter medications is urgently needed. Furthermore, reporting such adverse reactions to pharmacovigilance authorities is essential for epidemiological surveillance and the development of drug safety policies.

## Conclusions

TEN) is a potentially fatal condition with high mortality, particularly among elderly patients and those with comorbidities. This case illustrates the serious risks associated with the use of NSAIDs, including seemingly innocuous short-term exposures such as ibuprofen. The key to improving outcomes is early recognition, immediate withdrawal of the offending agent, intensive multidisciplinary supportive care, and systematic prognostic assessment. Emerging evidence supports the consideration of immunomodulatory agents such as cyclosporine in appropriate scenarios. The differential diagnosis of TEN is broad and includes several conditions that may mimic its clinical presentation, particularly widespread epidermal detachment and mucosal involvement. The most closely related condition is Stevens-Johnson Syndrome (SJS), which lies on the same clinical spectrum and differs primarily in the extent of body surface area involvement. Other relevant conditions include Staphylococcal Scalded Skin Syndrome (SSSS), which typically affects children, is characterized by more superficial epidermal cleavage, and lacks mucosal involvement; generalized bullous fixed drug eruption (GBFDE); autoimmune blistering diseases, such as pemphigus vulgaris and bullous pemphigoid; and systemic disorders, such as graft-versus-host disease (GVHD) and paraneoplastic pemphigus. Accurate diagnosis requires a detailed clinical evaluation, temporal correlation with drug exposure, histopathological examination, and, when indicated, direct immunofluorescence.

This case also underscores the urgent need for public education campaigns regarding the dangers of self-medication with commonly available analgesics and NSAIDs. The reporting of serious adverse drug reactions to national pharmacovigilance centers remains crucial to supporting public health strategies.
